# *Tautonia plasticadhaerens* sp. nov., a novel species in the family *Isosphaeraceae* isolated from an alga in a hydrothermal area of the Eolian Archipelago

**DOI:** 10.1007/s10482-020-01424-3

**Published:** 2020-05-12

**Authors:** Christian Jogler, Sandra Wiegand, Christian Boedeker, Anja Heuer, Stijn H. Peeters, Mareike Jogler, Mike S. M. Jetten, Manfred Rohde, Nicolai Kallscheuer

**Affiliations:** 1 Department of Microbiology, Radboud University, Nijmegen, The Netherlands; 2 Department of Microbial Interactions, Institute of Microbiology, Friedrich Schiller University, Jena, Germany; 3 Institute for Biological Interfaces 5, Karlsruhe Institute of Technology, Eggenstein-Leopoldshafen, Germany; 4 Leibniz Institute DSMZ, Brunswick, Germany; 5 Central Facility for Microscopy, Helmholtz Centre for Infection Research, Brunswick, Germany

**Keywords:** Marine bacteria, Panarea, Biotic surfaces, *Planctomycetes*, *Isosphaeraceae*, Hydrothermal vent system

## Abstract

A novel planctomycetal strain, designated ElP^T^, was isolated from an alga in the shallow hydrothermal vent system close to Panarea Island in the Tyrrhenian Sea. Cells of strain ElP^T^ are spherical, form pink colonies and display typical planctomycetal characteristics including division by budding and presence of crateriform structures. Strain ElP^T^ has a mesophilic (optimum at 30 °C) and neutrophilic (optimum at pH 7.5) growth profile, is aerobic and heterotrophic. It reaches a generation time of 29 h (µ_max_ = 0.024 h^−1^). The strain has a genome size of 9.40 Mb with a G + C content of 71.1% and harbours five plasmids, the highest number observed in the phylum *Planctomycetes* thus far. Phylogenetically, the strain represents a novel species of the recently described genus *Tautonia* in the family *Isosphaeraceae*. A characteristic feature of the strain is its tendency to attach strongly to a range of plastic surfaces. We thus propose the name *Tautonia plasticadhaerens* sp. nov. for the novel species, represented by the type strain ElP^T^ (DSM 101012^T^ = LMG 29141^T^).

## Introduction

The phylum *Planctomycetes,* along with *Chlamydiae*, *Verrucomicrobia* and others, forms the PVC superphylum, which is of environmental, medical and biotechnological importance (Spring et al. 2016; Wagner and Horn 2006). Members of the phylum *Planctomycetes* occur in a broad range of habitats on Earth, with the largest number of species so far isolated from aquatic environments (Wiegand et al. 2018). Phylogenetically, the phylum is subdivided into the classes *Phycisphaerae*, *Planctomycetia* and *Candidatus* Brocadiae. Recent rearrangements in the class *Planctomycetia* led to a more strictly defined order *Planctomycetales* and the introduction of the orders *Pirellulales*, *Gemmatales* and *Isosphaerales* (Dedysh et al. 2019). Species of the class *Planctomycetia* divide by budding, whereas members of the class *Phycipshaera*e divide by binary fission. Genome size ranges of 3–12 Mb and a G + C content of 40–71% have been observed in characterised strains of the phylum *Planctomycetes* (Ravin et al. 2018; Wiegand et al. 2020).

Strains clustering within *Planctomycetia*, the class with the currently highest number of characterised species in the phylum, have been shown to attach to various marine biotic surfaces, e.g. macroscopic phototrophs (Boersma et al. 2019; Bondoso et al. 2014, 2017; Peeters et al. 2020; Vollmers et al. 2017), on which they can be highly abundant (Bengtsson and Øvreås 2010). Such surfaces are suggested to serve as nutrient source, e.g. in the form of complex polysaccharides (Jeske et al. 2013; Lachnit et al. 2013). However, the survival of planctomycetal species appears counter-intuitive given their rather slow growth compared to natural competitors in this ecological niche (Frank et al. 2014; Wiegand et al. 2018). Strategies applied to compensate for lower growth rates may include the ability to produce bioactive secondary metabolites (Kallscheuer et al. 2019c; Panter et al. 2019), resistance against several antibiotics (Cayrou et al. 2010; Godinho et al. 2019) and/or a metabolism well-adapted to digestion of algae-derived compounds, including the above-mentioned polysaccharides. In this context, pili originating from conspicuous crateriform structures and an extremely enlarged periplasmic space observed in Planctomycetes may be involved in the uptake and intracellular cleavage of polymeric carbon sources, as shown for the model substrate dextran (Boedeker et al. 2017).

The cell envelope architecture of species of the phylum *Planctomycetes* was investigated based on super-resolution microscopic techniques and developed genetic tools (Jogler et al. 2011; Jogler and Jogler 2013; Rivas-Marin et al. 2016), which confirmed presence of peptidoglycan (Jeske et al. 2015; van Teeseling et al. 2015) and a cell envelope similar to that of Gram-negative bacteria (Boedeker et al. 2017; Devos 2014). However, in contrast to canonical bacteria, Planctomycetes lack otherwise essential divisome proteins, including FtsZ (Jogler et al. 2012; Pilhofer et al. 2008). In their genomes, 40–55% of the automatically annotated genes are of unknown function (Wiegand et al. 2020), which is a strong motivation to study the planctomycetal cell biology in greater detail.

To extend the collection of axenic cultures of Planctomycetes and as a basis for further study of their cell biology and metabolism, here we describe a novel strain, ElP^T^, isolated from an alga sampled in the Tyrrhenian Sea close to the island Panarea.

## Materials and methods

### Isolation of the novel strain and cultivation

For the isolation and cultivation of strain ElP^T^, M1H NAG ASW medium was used. Liquid and solid M1H NAG ASW medium was prepared as previously described (Boersma et al. 2019). Strain ElP^T^ was isolated from an alga gathered from hydrothermal area A26 (location: 38.6392 N 15.1051 E). With an average depth of 26 m, A26 is the deepest spot of a plateau located between the small islands Le Guglie and Lisca Bianca around 2.5 km east of the island Panarea, Italy. The geology of area A26 in the shallow-marine hydrothermal system close to Panarea is described elsewhere (Kürzinger 2019). Algal pieces were sampled on the 10th of September 2013 at a depth of 25 m and a water temperature of 19.4 °C. The sampled material was initially washed with sterile seawater containing 20 mg/L cycloheximide to prevent fungal growth. Afterwards, washed algal pieces were swabbed over a plate with M1H NAG ASW medium containing 8 g/L gellan gum, 1000 mg/L streptomycin, 200 mg/L ampicillin and 20 mg/L cycloheximide, which was subsequently incubated at 20 °C for four weeks. The 16S rRNA gene of the strains obtained was amplified by PCR with the primers 8f (5′-AGA GTT TGA TCM TGG CTC AG-3′) and 1492r (5′-GGY TAC CTT GTT ACG ACT T-3′) and then sequenced following a previously published protocol (Rast et al. 2017). This step was performed in order to check whether isolated strains represent members of the phylum *Planctomycetes*.

### Determination of pH and temperature optimum

The pH optimum and range were determined in M1H NAG ASW medium at 28 °C. The following buffers (each 100 mM) were used: 2-(*N*-morpholino)ethanesulfonic acid (MES) for pH 5.0 and 6.0, 4-(2-hydroxyethyl)-1-piperazineethanesulfonic acid (HEPES) for pH 7.0, 7.5 and 8.0, 3-(4-(2-hydroxyethyl)piperazin-1-yl)propane-1-sulfonic acid) (HEPPS) for pH 8.5 and *N*-cyclohexyl-2-aminoethanesulfonic acid (CHES) for pH 9.0 and 10.0. The temperature optimum and range were determined in standard M1H NAG ASW medium at pH 7.5. Growth was assessed by measuring the optical density at 600 nm (OD_600_). The average of OD_600_ values from three biological replicates was used for calculation of the growth rates. To this end, the natural logarithm of average OD_600_ values (ln(OD_600_)) was plotted against the cultivation time. The slope of the linear range of the curve (at least five data points) was used as maximal growth rate µ (in h^−1^). The generation time t_d_ (in h) was calculated using the equation t_d_ = ln(2)/µ.

### Microscopy protocols

Phase contrast and field emission scanning electron microscopy were performed as previously described (Boersma et al. 2019).

### Genome information and analysis of genome-encoded features

Genome and plasmid sequences of strain ElP^T^ are available from GenBank under accession numbers CP036426–CP036431. The 16S rRNA gene sequence of strain ElP^T^ can be found under accession number MK559970. DNA isolation and genome sequencing was carried out as part of a previous study (Wiegand et al. 2020). Numbers of carbohydrate-active enzymes were obtained from the CAZY database (Lombard et al. 2014). Gene clusters potentially involved in the production of secondary metabolites were determined using antiSMASH 4.0 (Blin et al. 2017).

### Phylogenetic analysis

16S rRNA gene sequence-based phylogeny was computed for strain ElP^T^, the type strains of all described planctomycetal species (assessed in January 2020) and all isolates published in the recent year (Boersma et al. 2019; Kallscheuer et al. 2019a, b, d; Kohn et al. 2019; Kovaleva et al. 2019; Peeters et al. 2020; Rensink et al. 2020) as previously described (Kallscheuer et al. 2019d). Three 16S rRNA genes of bacterial strains from the PVC superphylum, but outside of the phylum *Planctomycetes* (accession numbers AJ229235, NR_146840 and NR_027571), were used as the outgroup. The multi-locus sequence analysis (MLSA) was performed according to a previously published protocol (Kallscheuer et al. 2019d). The genomes of *Gemmata obscuriglobus* (accession number CP042911), *Rhodopirellula baltica* (accession number BX119912.1) and *Gimesia maris* (accession number CP043931) served as outgroup. The average nucleotide identity (ANI) was calculated using OrthoANI (Lee et al. 2016). The average amino acid identity (AAI) was obtained using the aai.rb script of the enveomics collection (Rodriguez-R and Konstantinidis 2016), while the percentage of conserved proteins (POCP) was calculated as described by Qin et al. (2014). The *rpoB* nucleotide sequences were taken from publicly available planctomycetal genome annotations and the sequence identities for the described 1200 bp sequence fragment were determined as previously described (Bondoso et al. 2013). Alignment and matrix calculation were performed with Clustal Omega (Sievers et al. 2011).

## Results and discussion

### Phylogenetic analysis

**Fig. 1:**
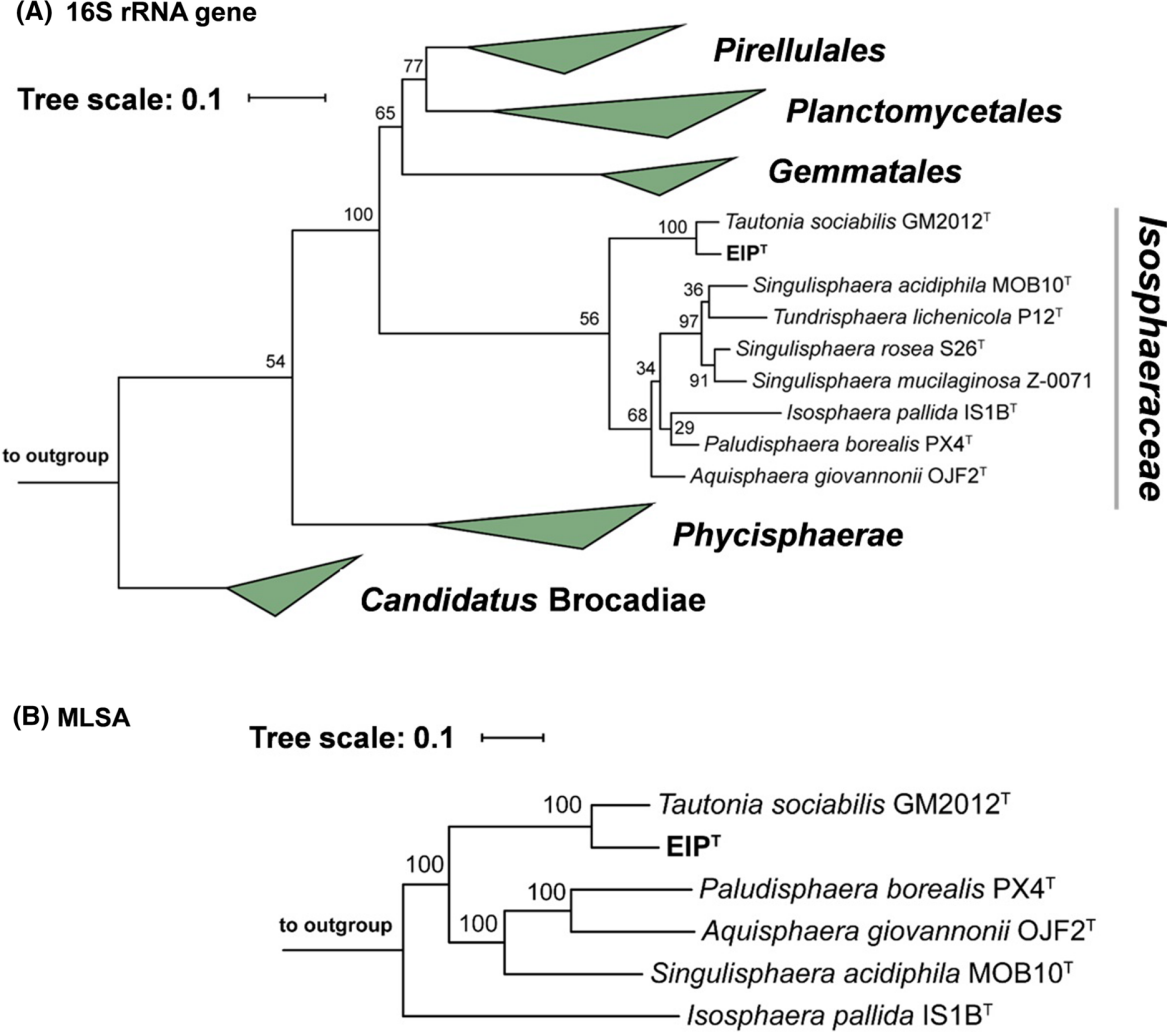
Maximum likelihood phylogenetic analysis. Phylogenetic trees showing the position of strain ElP^T^. 16S rRNA gene sequence-(**a**) and MLSA-based (**b**) phylogeny was computed as described in the “Materials and methods” section. Bootstrap values after 1000 re-samplings (16S rRNA gene sequences) and 500 re-samplings (MLSA) are given at the nodes (in %). The outgroups consist of three 16S rRNA genes from the PVC superphylum (16S rRNA-based tree) or the genome sequences of *Gemmata obscuriglobus*, *Rhodopirellula baltica* and *Gimesia maris* (MLSA-based tree)

**Fig. 2:**
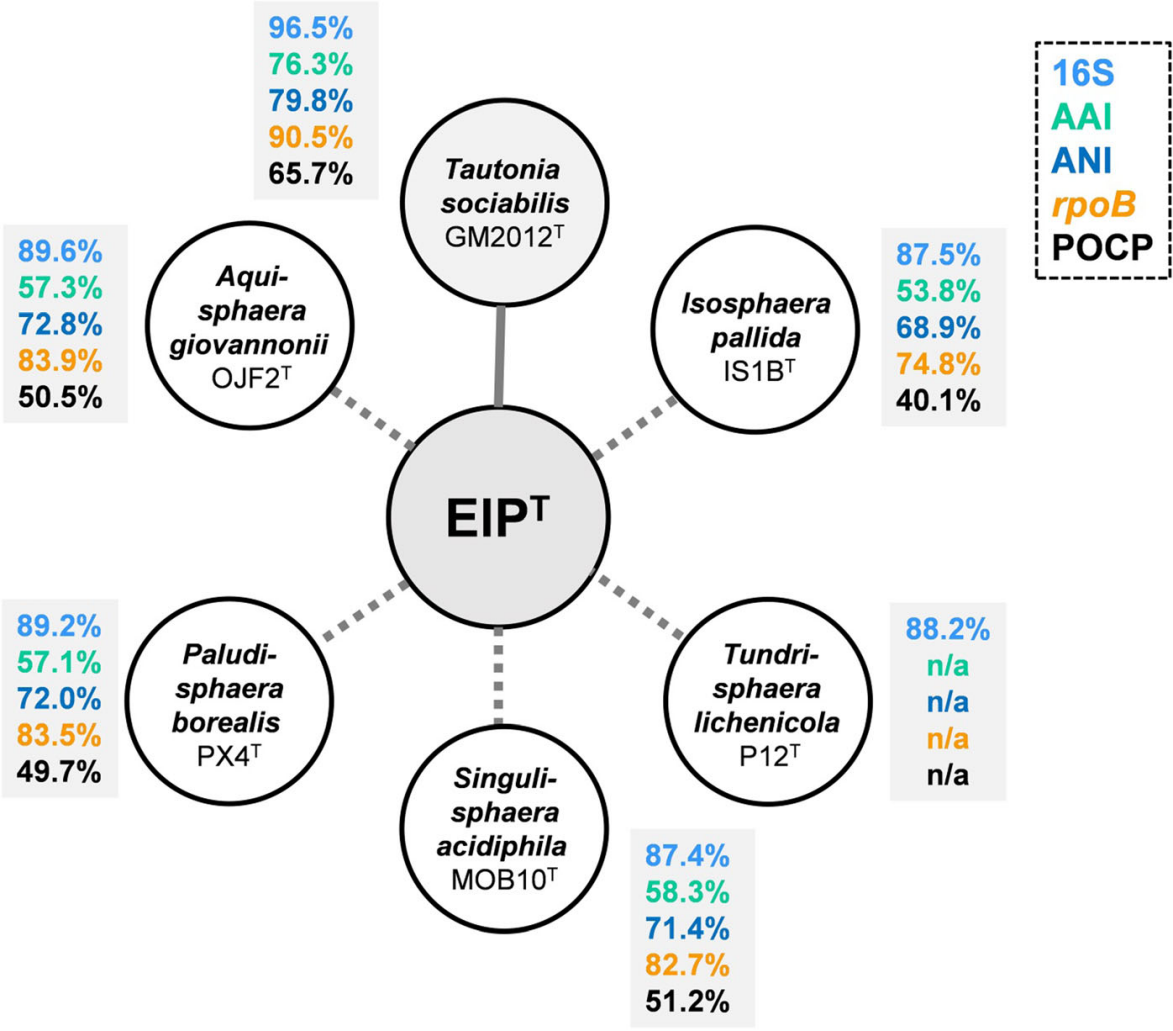
Similarity values of strain ElP^T^ in relation to species in the family *Isosphaeraceae*. Methods used: 16S rRNA gene sequence identity (16S), average amino acid identity (AAI), average nucleotide identity (ANI), *rpoB* gene identity (1200 bp fragment) and percentage of conserved proteins (POCP)

In both, the 16S rRNA gene sequence- and the MLSA-based phylogenetic tree (Fig. 1), strain ElP^T^ clusters monophyletically with *Tautonia sociabilis* GM2012^T^ (Kovaleva et al. 2019). The two strains share a 16S rRNA gene sequence similarity of 96.5% (Fig. 2), which is above the recommended genus threshold of 94.5%, but below the species threshold of 98.7% (Stackebrandt and Ebers 2006; Yarza et al. 2014). Comparison at the 16S rRNA gene level thus suggests that strain ElP^T^ represents a novel species in the genus *Tautonia*, family *Isosphaeraceae*. This finding is in line with an ANI of 79.8% obtained during comparison of strain ElP^T^ and *T. sociabilis*, since this value is below the threshold of 95% for strains belonging to the same species (Kim et al. 2014). For a more extensive evaluation, additional phylogenetic markers were taken into account. Indeed, affiliation of strain ElP^T^ to the genus *Tautonia* and simultaneous delineation from *T. sociabilis* is supported by AAI, *rpoB* similarity and POCP values of 76.3%, 90.5% and 65.7%, respectively (Fig. 2). These values fall above the recommended genus thresholds of 60–80% (AAI), 75.5–78% (*rpoB*) and 50% (POCP) for delineation of prokaryotic genera, but below the thresholds of 95% (AAI) and 96.3% (*rpoB*) for differentiation of species (Kallscheuer et al. 2019d; Konstantinidis and Tiedje 2005; Qin et al. 2014). *T. sociabilis* was clearly established as the current closest relative of strain ElP^T^ since lower similarity values were obtained for comparison with species of other known genera in the family *Isosphaeraceae*, namely *Isosphaera*, *Singulisphaera*, *Aquisphaera*, *Paludisphaera* and *Tundrisphaera*. For comparison of strain ElP^T^ with species of the mentioned genera, AAI and 16S rRNA gene similarity values are below the genus threshold, while in most cases the POCP was found to be at or slightly above the genus threshold of 50% (Fig. 2). Similarity of *rpoB* is used as phylogenetic marker in the order *Planctomycetales* (Bondoso et al. 2013) and a genus threshold of 75.5–78% was recently proposed based on new strains in the family *Pirellulaceae* (former members of *Planctomycetaceae*) (Kallscheuer et al. 2019d). Based on the obtained values (Fig. 2), the *rpoB* genus threshold is probably not applicable to the family *Isosphaeraceae*.

### Morphological and physiological analyses

**Fig. 3:**
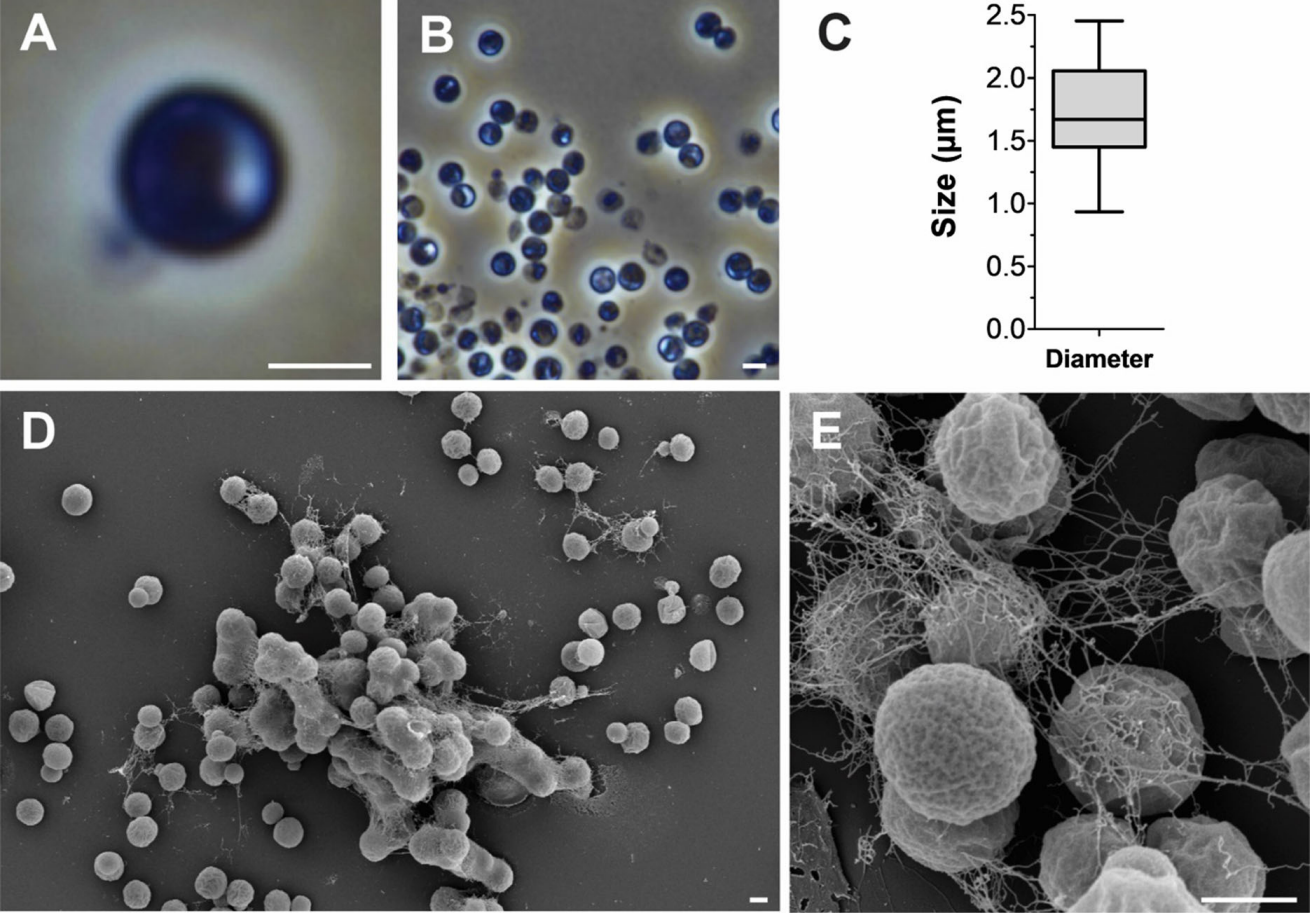
Microscopy images and cell size plot of strain ElP^T^. The mode of cell division (**a**) and a general overview of the cell morphology (**b**, **d**, **e**) is shown. Cells tend to form aggregates surrounded by an extracellular matrix (**d** + **e**). The scale bar is 1 µm. For determination of the cell size (**c**) at least 100 representative cells were counted manually or by using a semi-automated object count tool

Basic features of strain ElP^T^ comprising cell morphology, growth and mechanism of cell division are summarised in Table 1 and compared to *T. sociabilis*, *Isosphaera pallida*, *Tundrisphaera lichenicola*, *Singulisphaera acidiphila*, *Paludisphaera borealis* and *Aquisphaera giovannonii* (Bondoso et al. 2011; Giovannoni et al. 1987; Kovaleva et al. 2019; Kulichevskaya et al. 2008, 2016, 2017). Morphological features of ElP^T^ cells harvested during the exponential growth phase were analysed using phase contrast and scanning electron microscopy (Fig. 3). Strain ElP^T^ forms spherical cells with a typical diameter of 1.4–2.0 µm (Fig. 3a, c), which occur either as single cells or form smaller aggregates of 25–40 cells. Cells divide by budding with the bud having the same shape as the mother cell (Fig. 3a). Cell size and shape of ElP^T^ are comparable to the type species of known genera in the family *Isosphaeraceae*, with the exception of *I. pallida*, which forms cells that are considerably larger. All seven compared strains follow the same mode of division and contain crateriform structures on the entire cell surface (no data available for *T. sociabilis*). The colonies of ElP^T^ have a pink pigmentation, suggesting the production of carotenoids. The colour is similar to the species chosen for comparison, with the exception of *S. acidiphila* and the closely related *T. sociabilis*, which lack pigmentation (Table 1). Once isolated, colonies of strain ElP^T^ were observed to grow at the plastic boundary of the Petri dish, only half connected to the agar surface. This tendency to stick to plastic surfaces necessitated the use of glassware for handling of the strain. For example, cells stuck strongly to plastic pipettes, making their transfer difficult. This immediate adsorption towards plastic surfaces might be related to the extracellular matrix abundantly produced by strain ElP^T^ (Fig. 3e). The strain was determined to be non-motile as are the other reference species, except *I. pallida*, which displays phototactic gliding motility (Giovannoni et al. 1987).

**Table 1:** Phenotypic and genotypic features of strain ElP^T^ compared to closely related strains. The genome analysis is based on GenBank accession numbers for strain ElP^T^ (CP036426–CP036431), *Tautonia sociabilis* (GCA_003977685.1), *Isosphaera pallida* (CP002353–CP002354), *Singulisphaera acidiphila* (CP003364–CP003367), *Paludisphaera borealis* (CP019082–CP019084) and *Aquisphaera giovannonii* (CP042997–CP042999). The genome of *Tundrisphaera lichenicola* has not been sequenced yet

Feature	ElP^T^	*Tautonia sociabilis* GM2012^T^	*Isosphaera pallida* IS1B^T^	*Tundrisphaera lichenicola* P12^T^	*Singulisphaera acidiphila* MOB10^T^	*Paludisphaera borealis* PX4^T^	*Aquisphaera giovannonii* OJF2^T^
*Phenotypic characteristics*							
Shape	Spherical	Spherical	Spherical	Spherical	Spherical	Spherical	Spherical
Diameter (µm)	1.7 ± 0.3	1.7–2.9	2.5–3.0	2.2–3.0	1.6–2.6	1.5–2.5	1.6–2.0
Colour	Pink	White	Pink	Pink	White	Bright pink	Pink
Relation to oxygen	Aerobic	Strictly aerobic	Strictly aerobic	Strictly aerobic	Strictly aerobic	Aerobic	Strictly aerobic
Temperature range (optimum) (°C)	10–33 (30)	37–46 (42)	34–55 (41)	4–28 (15–22)	4–33 (20–26)	6–30 (15–25)	10–35 (30)
pH range (optimum)	6–0-8.5 (7.5)	5.5–9.0 (7.5)	7.8–8.8	4.5–6.8 (5.5–6.0)	4.2–7.5 (5.0–6.2)	3.5–6.5 (5.0.-5.5)	6.5–9.5 (7.5–8.5)
Division	Budding	Budding	Budding	Budding	Budding	Budding	Budding
Dimorphic life cycle	n.o.	n.d.	n.d.	n.d.	n.d.	n.d.	n.d.
Motility	No	No	Yes, phototactic gliding	No	No	No	No
Crateriform structures	Ubiquitous	n.d.	Ubiquitous	Ubiquitous	Ubiquitous	Ubiquitous	Ubiquitous
Fimbriae	Yes	n.d.	Yes	n.d.	n.d.	n.d.	Yes
Capsule	Yes	n.d.	No	n.d.	Yes	n.d.	Yes
Stalk	n.o.	n.d.	n.d.	No	No	n.d.	n.d.
Holdfast structure	n.o.	n.d.	No	Yes	Yes	Yes	n.d.
*Genomic characteristics*							
Genome size (bp)	9,395,224	6,760,005	5,529,304	n.d.	9,755,686	7,651,896	10,526,296
Plasmids	5	n.d.	1	n.d.	3	2	2
G + C (%)	71.1 ± 0.8	70.1	62.5 ± 3.2	61.2–62.2	62.2 ± 2.3	66.3 ± 4.1	70.8 ± 0.5
Coding density (%)	84.7	85.0	84.7	n.d.	83.5	86.1	85.7
Completeness (%)	98.28	98.28	98.28	n.d.	98.28	96.55	96.55
Contamination (%)	5.17	3.45	0	n.d.	6.90	3.45	5.17
Total genes	7707	5183	3828	n.d.	7689	5961	7953
Genes/Mb	820	767	692	n.d.	788	779	756
Giant genes	0	0	0	n.d.	1	0	1
All protein-coding genes	7556	5084	3761	n.d.	7540	5855	7835
Protein-coding genes/Mb	804	752	680	n.d.	773	765	744
Hypothetical proteins	3399	3175	1821	n.d.	4316	3154	3328
tRNAs	100	84	51	n.d.	81	83	107
16S rRNA genes	3	1	3	n.d.	8	3	3

*n.o.* not observed, *n.d.* not determined

**Fig. 4:**
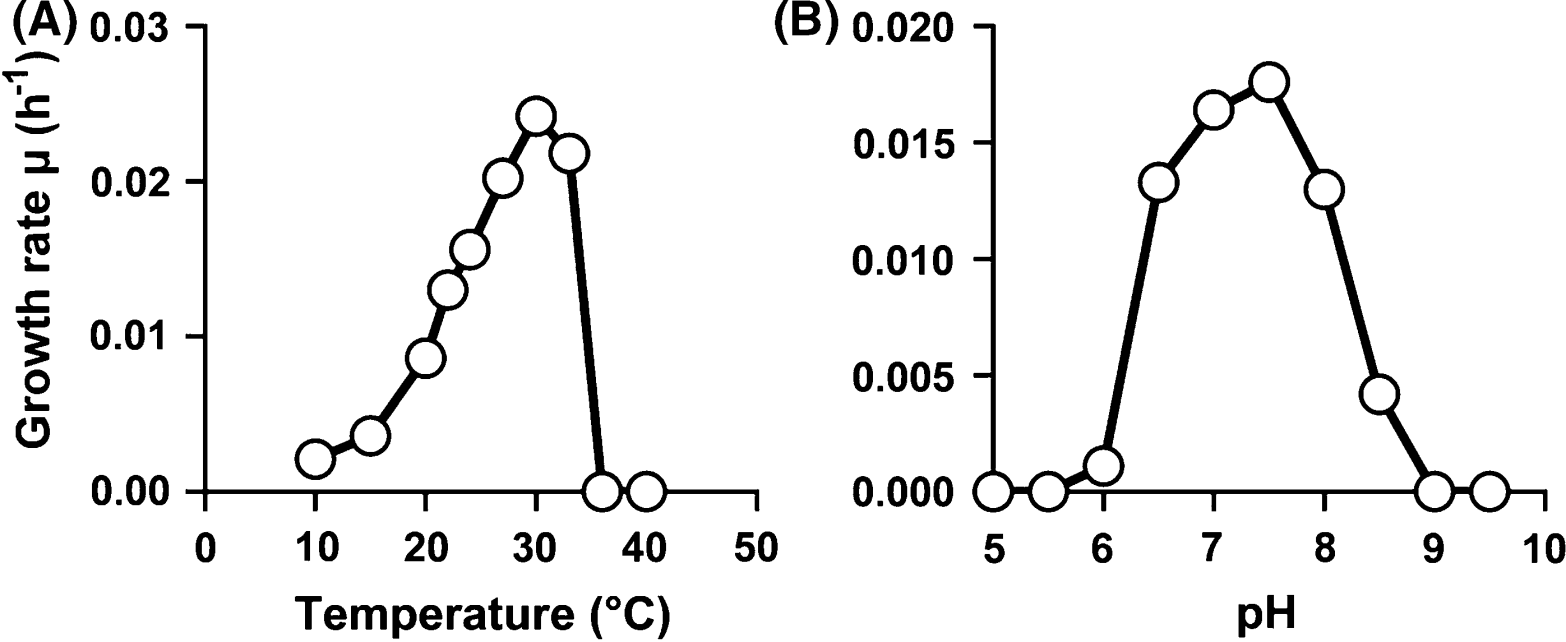
Temperature and pH optimum of ElP^T^. The graphs show the average growth rates obtained from cultivation of the strain in M1H NAG ASW medium in biological triplicates. Cultivations at different temperatures (**a**) were performed at pH 7.5 and cultivations at different pH values (**b**) were conducted at 28 °C

In M1H NAG ASW medium, strain ElP^T^ was able to grow over a temperature range of 10–33 °C and a pH range of 6.0–8.5 (Fig. 4). Strain ElP^T^ was found to be aerobic, heterotrophic, mesophilic and neutrophilic. Optimal growth was observed at 30 °C and pH 7.5, which led to a maximal growth rate of 0.024 h^−1^, corresponding to a generation time of 29 h (Fig. 4). The family *Isosphaeraceae* appears to be heterogeneous regarding temperature and pH preferences. *S. acidiphila, T. lichenicola* and *P. borealis* favour lower temperatures (15–26 °C) compared to strain ElP^T^ (30 °C), whereas *I. pallida* and *T. sociabilis* are thermophiles with optimal growth at 41–42 °C and a temperature range allowing growth up to 55 °C (Table 1). With regard to pH, *I. pallida* and *A. giovannonii* are adapted to slightly alkaline growth conditions (pH 8–9), whereas *S. acidiphila, T. lichenicola* and *P. borealis* require more acidic environments (pH 5–6). Strain ElP^T^ and *T. sociabilis* grow optimally under neutral conditions (pH 7–7.5). These differences likely reflect the different natural habitats from which the strains were isolated. *I. pallida* was isolated from a hot spring, explaining the preference for higher temperatures, while e.g. *S. acidiphila* was isolated from a *Sphagnum* peat moss, which are typically found in nutrient-poor and acidic peat bogs (Giovannoni et al. 1987; Kulichevskaya et al. 2008).

### Genomic characteristics

The complete genome of strain ElP^T^ has a size of 9.40 Mb, distributed among the chromosome (8.67 Mb) and five plasmids (with sizes of 0.28, 0.14, 0.12, 0.09 and 0.09 Mb). While plasmids are scarce among Planctomycetes, the family *Isosphaeraceae* is exceptional in that regard (Ivanova et al. 2017). Five strains of this family harbour at least one plasmid (no data available for *T. sociabilis*), while four plasmids was the current maximum observed in strain SH-PL62 (Ivanova et al. 2017). Strain ElP^T^ maintains five extrachromosomal replicons in parallel, making it a potential resource for future planctomycetal genetic tool development.

The G + C content of strain ElP^T^ is 71.1%. In its genome, 7707 genes were annotated, of which 7556 are putative protein-coding genes. The number of hypothetical proteins is 3399, corresponding to 45% of the total number of putatively annotated proteins. The number of protein-coding genes yields 804 encoded proteins per Mb and a coding density of 84.7%. 100 tRNAs and three copies of the 16S rRNA gene were identified. The genomic characteristics of the compared strains are quite heterogeneous (Table 1). With 5.53 Mb *I. pallida* has by far the smallest genome, while the genomes of strain ElP^T^, *S. acidiphila* and *A. giovannonii* fall in a size range of 9.4–10.6 Mb. Their G + C content varies from 62 to 71%, with strain ElP^T^ showing the highest G + C content of the compared strains. One giant gene (> 5 kb) was found in *S. acidiphila* and *A. giovannonii*, while the other three strains lack giant genes. The genome of *T. lichenicola* has not been sequenced yet and could thus not be used for comparison.

### Genome-based analysis of the primary and secondary metabolism

The genome sequences of species belonging to the family *Isosphaeraceae* provide important information on their metabolic capabilities. The suggested capability of Planctomycetes to degrade high molecular weight sugars is likely reflected by high numbers of carbohydrate-active enzymes encoded in their genomes, while production of secondary metabolites is often related to interactions with the abiotic and biotic environment, including response to external stress factors. The compared members of the family *Isosphaeraceae* harbour between 109 and 317 carbohydrate-active enzymes and a clear correlation between the number of enzymes and the genome size was observed (Table 2). Only *S. acidiphila* slightly deviates from this trend. It has the second largest genome of the compared species, but is only ranked 3rd with regard to the number of carbohydrate-active enzymes. Strain ElP^T^ has a 5% smaller genome, but its number of carbohydrate-active enzymes is around 10% higher. Analysis of the distribution to the different enzyme families shows that glycoside hydrolases and glycosyl transferases account for 80–90% of the total number in all five strains. *A. giovannonii* has a considerably higher number of enzymes of the glycoside hydrolase family, which is 2.5 times as high as in strain ElP^T^ (second highest number of enzymes of this family) and almost seven times as high as in *I. pallida*. Whether a higher number of carbohydrate-active enzymes is related to a higher versatility during degradation naturally-occuring polysaccharides remains to be elucidated.

**Table 2:** Numbers of carbohydrate-active enzymes and secondary metabolite-associated gene clusters in ElP^T^ in comparison to other species in the family *Isosphaeraceae*. The analysis is based on GenBank accession numbers for strain ElP^T^ (CP036426–CP036431), *Tautonia sociabilis* (GCA_003977685.1), *Isosphaera pallida* (CP002353–CP002354), *Singulisphaera acidiphila* (CP003364–CP003367), *Paludisphaera borealis* (CP019082–CP019084) and *Aquisphaera giovannonii* (CP042997–CP042999)

Feature	ElP^T^	*Tautonia sociabilis* GM2012^T^	*Isosphaera pallida* IS1B^T^	*Singulisphaera acidiphila* MOB10^T^	*Paludisphaera borealis* PX4^T^	*Aquisphaera giovannonii* OJF2^T^
Genome size (Mb)	9.40	6.76	5.53	9.76	7.65	10.53
*Carbohydrate-active enzymes*						
Glycoside Hydrolase Family	59	n.d.	21	49	52	142
Glycosyl Transferase Family	123	n.d.	74	117	86	120
Polysaccharide Lyase Family	3	n.d.	2	1	0	3
Carbohydrate Esterase Family	13	n.d.	5	9	9	17
Carbohydrate-Binding Module Family	16	n.d.	7	14	21	35
Total number	214	n.d.	109	190	168	317
*Secondary metabolite-associated clusters*						
Terpenoid	3	2	3	3	2	2
Type I Polyketide synthase	1	1	1	2	3	2
Type II Polyketide synthase	0	0	0	0	0	0
Type III Polyketide synthase	0	0	1	0	1	1
Non-ribosomal peptide synthetase	0	0	0	0	0	1
Bacteriocin	0	2	0	1	0	1
Resorcinol	0	0	0	0	0	0
Total number	4	5	5	6	6	7

To gain a first insight into the secondary metabolism, numbers of genes coding for polyketide synthases (PKSs), non-ribosomal peptide synthetases (NRPSs) and other genes involved in the synthesis of terpenoids, bacteriocins or resorcinol were analysed (Table 2). A correlation between the number of gene clusters and the genome size could also be observed in this case. Five genes/gene clusters were found in species with genome sizes of 5–7 Mb, six in species with 7–10 Mb and seven clusters in *A. giovannonii* with > 10 Mb genome size. Strain ElP^T^ is an exception to this trend since only four clusters were observed, although the strain has the second largest genome of those compared. All six strains harbour 2–3 genes putatively involved in terpenoid biosynthesis. Genes coding for phytoene synthase isoenzymes (CrtB; catalysing the initial step during carotenoid biosynthesis) were identified in the pink-pigmented strains (see Table 1), however, genes in *T. sociabilis* and *S. acidiphila* might well code for closely related squalene synthases. Since the pathway for carotenoid biosynthesis in Planctomycetes has not been discovered yet, additional conclusions cannot be drawn from the genome sequence at this stage. At least one type I PKS-encoding gene is present in all six strains, while three of the strains also harbour a putative type III PKS gene. Type II PKSs were not observed in the compared strains. Two of the strains appear to be capable of bacteriocin production, while a single NRPS-encoding gene was observed in *A. giovannonii*. The six species may harbour additional gene clusters involved in the production of small molecules, these, however, might have escaped the in silico prediction by the AntiSMASH tool.

Taken together, comparison of morphological, physiological and genomic features in the heterogeneous family *Isosphaeraceae* supports the results of the phylogenetic analysis, which leads us to the conclusion that strain ElP^T^ represents a novel species in the genus *Tautonia*. Thus, we propose the name *Tautonia plasticadhaerens* for this species, represented by the type strain ElP^T^ (DSM 101012^T^ = LMG 29141^T^).

### Emended genus description of *Tautonia* Kovaleva et al. (2019)

The description of the genus is as previously published (Kovaleva et al. 2019), with the following modification: species of this genus are mesophilic or thermotolerant.

### *Tautonia plasticadhaerens* sp. nov.

Plas.tic.ad.hae'rens. N.L. neut. n. *plasticum* plastic; L. pres. part. *adhaerens* adhering, sticking to; N.L. part. adj. *plasticadhaerens *attaching to plastic, due to the tendency of the type strain to attach strongly to plastic surfaces.

Cells are spherical (diameter 1.7 ± 0.3 µm), occur as single cells or small aggregates and divide by budding. Stalk-free and non-motile cells, which contain crateriform structures covering the entire cell surface. Cells produce an extracellular matrix and strongly attach to plastic surfaces. Colonies are pink. Cells of the type strain grow over a temperature range of 10–33 °C (optimum 30 °C) and at pH 6.0–8.5 (optimum 7.5). The genome of the type strain has a size of 9.40 Mb, which is distributed among the chromosome and five plasmids. The G + C content is 71.1%.

The type strain is ElP^T^ (DSM 101012^T^ = LMG 29141^T^), isolated from an alga close to Panarea Island in September 2013.
